# 249. Quantifying the Relationship Between Renal Function and Procalcitonin: A Study of 14,431 Blood Cultures

**DOI:** 10.1093/ofid/ofaf695.092

**Published:** 2026-01-11

**Authors:** Albert Park, Trisha S Nakasone, Amit Kaushal, Cybele Renault

**Affiliations:** Stanford Medicine, San Mateo, CA; Veterans Affairs Palo Alto Health Care System, Palo Alto, California; Veterans Affairs Palo Alto Health Care System, Palo Alto, California; Veterans Affairs Palo Alto Health Care System, Palo Alto, California

## Abstract

**Background:**

Procalcitonin (PCT) is a potential biomarker for bloodstream infections. While reduced reliability in renal dysfunction has been noted, this relationship remains poorly quantified. We aimed to quantify the association between estimated glomerular filtration rate (eGFR) and PCT positivity in suspected bacterial bloodstream infections.

**Table 1.**
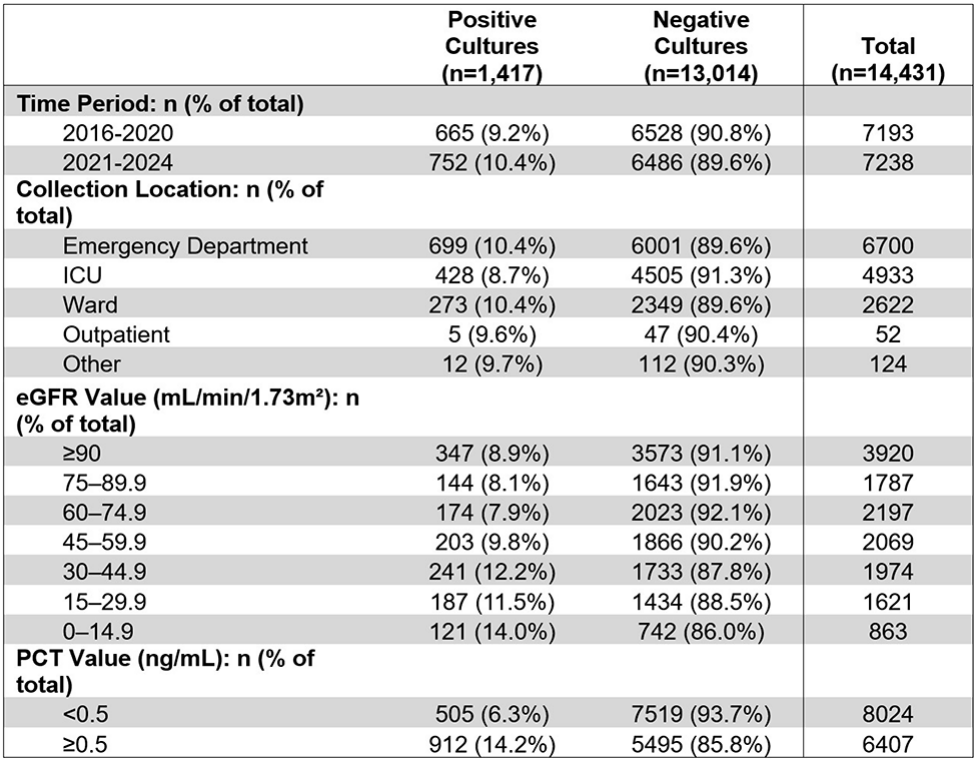
Blood culture characteristics. SD, standard deviation; ICU, intensive care unit; PCT, procalcitonin; eGFR, estimated glomerular filtration rate.

**Figure 1.**
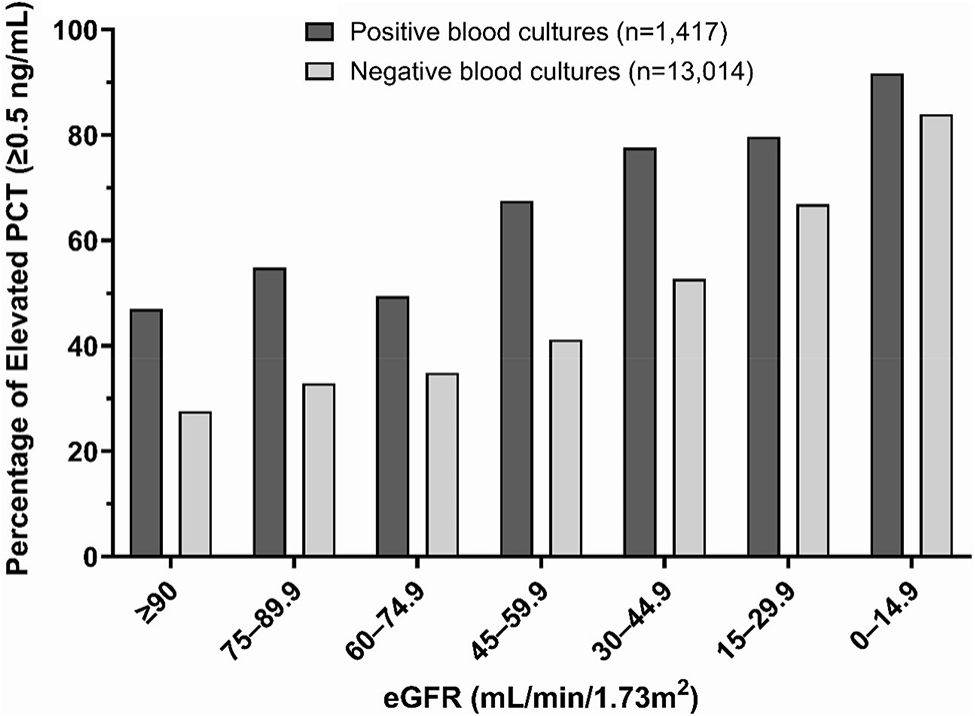
Percentage of positive procalcitonin values stratified by eGFR categories in positive and negative blood cultures. Bar graphs show the percentage of PCT values ≥0.5 ng/mL across eGFR categories for positive (dark gray, n=1,417) and negative (light gray, n=13,014) blood cultures. PCT, procalcitonin; eGFR, estimated glomerular filtration rate.

**Methods:**

In this retrospective study, we analyzed 14,431 blood cultures from 2,832 patients (2016-2024). We identified PCT values within 48 hours of culture collection with corresponding same-day eGFR. Cultures were classified as positive/negative based on bacterial identification. We categorized eGFR into seven clinical categories (0-14.9 to ≥90 mL/min/1.73m²). Using multivariate regression, we assessed the relationship between eGFR and PCT positivity (≥0.5 ng/mL), adjusting for age and race. We evaluated whether this relationship differed between positive/negative cultures and performed sensitivity analyses across multiple PCT thresholds.

**Table 2.**
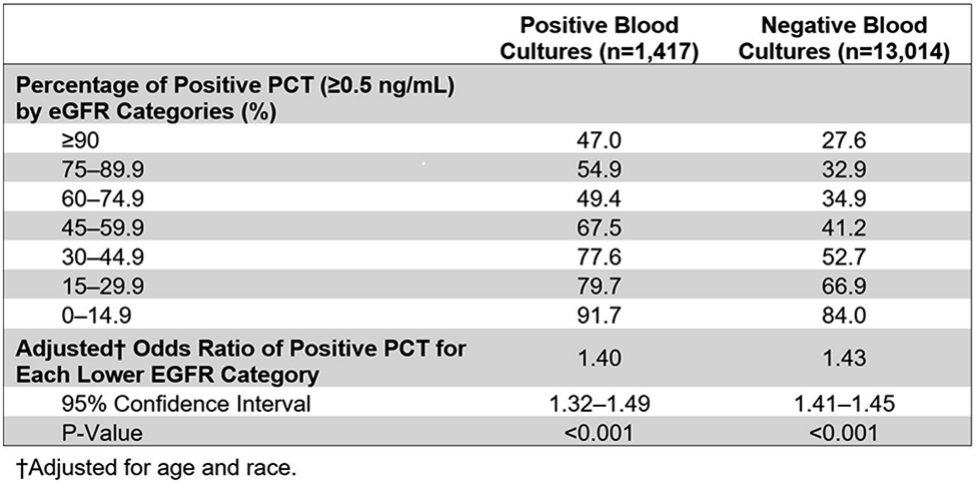
EGFR-stratified procalcitonin levels in positive and negative blood cultures. PCT, procalcitonin; eGFR, estimated glomerular filtration rate.

**Results:**

Of 14,431 cultures, 9.8% were positive (Table 1). PCT positivity increased with declining renal function (Figure 1). In positive cultures, PCT positivity increased from 47.0% with normal function (eGFR ≥90) to 91.7% in severe dysfunction (eGFR < 15) (Table 2). In negative cultures, this proportion increased from 27.6% to 84.0%. For each category of decreased eGFR, the odds of PCT positivity were approximately 40-43% higher (adjusted OR 1.40, 95% CI: 1.32-1.49 for positive cultures; 1.43, 95% CI: 1.40-1.46 for negative cultures; both p< 0.001).

**Conclusion:**

This analysis demonstrated an inverse relationship between renal function and PCT positivity. Each category of worsening renal function increased the odds of a positive PCT by approximately 40-43%, regardless of whether a bloodstream infection was present. This relationship remained consistent even when adjusting for age and race. Traditional PCT cutoffs became less reliable as renal function worsened, particularly with eGFR < 30, where PCT positivity exceeded 65% regardless of culture status. These findings suggest PCT values should be interpreted cautiously in renal dysfunction, as the 0.5 ng/mL cutoff may not reliably distinguish between presence and absence of infection.

**Disclosures:**

All Authors: No reported disclosures

